# Hepatoma upregulated protein expression is involved in the pathogenesis of human breast carcinogenesis

**DOI:** 10.3892/ol.2014.2614

**Published:** 2014-10-13

**Authors:** JIN CHEN, QIU-JUN LIU, DA WANG, XIAN-YAO ZHOU, DING XIONG, HONG-JIANG LI, CHANG-LONG LI

**Affiliations:** 1 Department of Biochemistry and Molecular Biology, Sichuan University, Chengdu, Sichuan 610072, P.R. China; 2 Department of Thyroid and Breast Surgery, West China Hospital of Sichuan University, Chengdu, Sichuan 610072, P.R. China

**Keywords:** hepatoma upregulated protein expression, breast carcinogenesis, cell proliferation

## Abstract

In the last decade, the overexpression of hepatoma upregulated protein (HURP) has been reported in hepatocellular carcinoma, adrenocortical tumors and urogenital carcinoma. However, the role of HURP in breast cancer remains unknown. In the present study, a comprehensive analysis was performed to examine the HURP expression level in 43 breast cancer tumor samples and paired adjacent normal tissues. The correlation between the HURP expression level and the clinicopathological characteristics was evaluated. The role of HURP in breast cancer was investigated by quantitative polymerase chain reaction, western blot analysis and cell proliferation assays. HURP expression was found to be significantly increased in the breast cancer samples. The HURP expression level was higher in the tumors with advanced-grade metastasis and was strongly associated with tumor-node-metastasis staging (P=0.003). Transfection and cell proliferation assays suggested that the suppression of HURP expression or the interference in HURP activity in the breast cancer cells inhibited cell proliferation significantly. These data suggest that HURP is associated with the degree of malignancy and the proliferation of breast cancer. HURP could be a tumor biomarker for prognosis and a potential therapeutic drug target for human breast cancer.

## Introduction

Female breast cancer accounts for one in 10 of all new cancer cases diagnosed each year worldwide. As the most prevalent cancer amongst females in developing and developed countries, breast cancer is the leading global cause of female cancer-related mortality (1,2). Although improvements in the early detection and treatment of breast cancer have decreased mortality rates in recent years, the survival rates for patients with late-stage or metastatic breast cancer remain poor (3). Thus, it is important to identify novel genes or pathways involved in breast cancer to develop faster diagnoses and safer treatments.

Hepatoma upregulated protein (HURP) was initially classified as an upregulated protein in human hepatocellular carcinoma and was demonstrated to be an integral part of the spindle apparatus (4,5). Further studies have revealed HURP to be a novel component of the Ran-importin β-regulated spindle assembly pathway, which forms a complex with guanosine-5′-triphosphate (RanGTP) and localizes predominantly to the kinetochore microtubules (K-MTs) supporting kinetochore fiber (k-fiber) stabilization (6,7). HURP is also a mitotic phosphoprotein substrate for Aurora-A, a mitotic serine/threonine kinase with oncogenic properties (8,9). Spindle assembly and function are controlled by the phosphorylation of HURP by Aurora-A, which acts as a regulatory mechanism (10). HURP abundance is tightly regulated during the cell cycle, with the levels of HURP fluctuating during the cycle and reaching a peak at G_2_/M (5). This fact suggests that HURP is a potential cell cycle regulator. HURP promotes chromosome congression and controls spindle stability by combining with k-fibers. HURP activity is necessary for correct kinetochore capture, effective chromosome congression and prompt mitotic progression. Defects in these regulatory process can lead to mitotic delay, misaligned chromosomes and genomic instability (6,9–11). As genomic instability is a noteworthy feature of human cancer (12,13), we hypothesized that HURP may have a role in the progression of breast carcinogenesis.

The overexpression of HURP has thus far been identified in hepatocellular carcinoma, adrenocortical tumors and urogenital carcinoma (14,15). However, there is no information about HURP in human breast carcinogenesis progression and the clinical relevance of HURP in cancer patients. In the present study, the clinicopathological and functional activities of HURP in human breast carcinogenesis were investigated. The present findings demonstrate the significance of the overexpression of HURP in human breast carcinogenesis and its functional role *in vitro*.

## Material and methods

### Patients and specimen collection

In total, 43 breast cancer tumor samples and paired normal tissues were obtained from patients who underwent surgery in West China Hospital (Sichuan University, Chengdu, China) between 2011 and 2012. The normal tissue was extracted at least 5 cm distal from the primary breast cancer and was identified as normal by a pathologist. All specimens were immediately frozen in liquid nitrogen and stored at −80°C until RNA was extracted. All patients provided written informed consent and the procedures were approved by the Human Ethics Review Board. No patients received chemotherapy or radiotherapy prior to surgery. All demographic and pathological data, including the patient age, tumor size and stage, number of tumors, presence of lymph node metastasis, immunohistochemical results and histological classification were obtained from clinical and pathological database records. All specimens were graded using a modification of the World Health Organization classification system (16), and the pathological staging was performed according to the pathological tumor-node-metastasis (TNM) staging system (17).

### RNA preparation, reverse transcription (RT) and quantitative polymerase chain reaction (qPCR)

Total RNA was extracted from frozen tissue specimens and cells using TRIzol reagent (Life Technologies, Gaithersburg, MD, USA). cDNA was then synthesized with a PrimeScript RT reagent kit with gDNA Eraser (Takara Biotechnology (Dalian) Co., Ltd., Dalian, China). The sequences of the HURP primers were designed as follows: Sense, 5′-CAT GTGAAGAAGACTTTGTTTTTGA-3′; and antisense, 5′-GGTAATCCAGGACACTGAGCA-3′. The glyceraldehyde-3 phosphate dehydrogenase (GAPDH) gene served as an internal quality RNA reference control. The sequences of the GAPDH primers were as follows: Sense, 5′-ACCACAGTCCATGCCATCAC-3′; and antisense, 5′-TCCACCACCCTGTTGCTGTA-3′. qPCR was performed in MyiQTM and iQTM5 Real-Time PCR Detection Systems (Bio-Rad Laboratories, Hercules, CA, USA) using the SsoFast™EvaGreen^®^Supermix (Bio-Rad Laboratories). qPCR was performed as follows: Enzyme activation at 95°C for 30 sec, followed by 40 cycles of denaturation at 95°C for 5 sec and annealing at 55°C for 5 sec. All mRNA copy numbers were calculated relative to the concentration of cDNA from Human Universal Reference total RNA (Takara Bio, Inc., Shiga, Japan). The HURP copy number was then divided by the copy number of the endogenous reference (GAPDH) to obtain normalized expression values.

### HURP protein expression analysis

Total protein was extracted from the specimens and cells using RIPA Lysis Buffer (YuanPingHao Bio, Beijing, China). Aliquots of total protein were separated on 10% acrylamide gradient gels. Following electrophoresis, the samples were electroblotted (45 mA, 90 min) onto a polypropylene difluoride membrane (Millipore, Billerica, MA, USA). Anti-HURP rabbit polyclonal antibody (Ab; Santa Cruz Biotechnology, Inc., CA, USA) detected HURP protein at a 1:200 dilution. The protein level of HURP was normalized to the level of GAPDH protein, which was detected by a 1:10,000 dilution of GAPDH rabbit monoclonal (Mc)Ab (Epitomics, Inc., Burlingame, CA, USA). Following incubation with a secondary Ab, peroxidase-conjugated Affinipure goat anti-rabbit immunoglobulin G (ZSGB-Bio, Beijing, China) at a dilution of 1:5,000, the protein signals were visualized by chemiluminescence (Pierce, Rockford, IL, USA). The intensity of the bands was measured by Quantity One Software (Bio-Rad) and normalized using the intensity of GAPDH.

### Cell lines and cell culture

In total, four human breast cancer cell lines, MCF-7, MDA-MB-231, MDA-MB-435S and ZR-75-30, were obtained from Zhengzhou Jinrong Biotechnology Co., Ltd., (Zhengzhou, Henan, China) and were routinely maintained in RPMI-1640 with 10% (vol/vol) fetal bovine serum at 37°C, in a humidified atmosphere of 95% air and 5% CO_2_.

### Small interfering (si)RNA

Gene-specific 27mer siRNA duplexes (DsiRNAs) designed to target the HURP gene (DsiRNA1, rArGrArCrCrArGrUrArCrArGrGrArT; DsiRNA2, rCrCrUrArUrCrArArGrUrArArCrArCrCrUrArUrGrArCrUCC; and DsiRNA3, rArCrCrUrArArGrUrCrUrGrUrCrArArCrArArArGrCrUrGTA) were obtained from a Trilencer-27 siRNA kit (OriGene, Rockville, MD, USA), and universal scrambled negative control siRNA duplex (OriGene) was used as a negative control. The cells (1.2×10^5^) were transfected with a 10-nM final concentration of the respective siRNAs, using a siTRAN (OriGene) for 24 h to harvest the cells and detect the mRNA levels in the parental, negative and HURP siRNA cells, according to the manufacturer’s instructions.

### Protein transfection

The Xfect Protein Transfection Reagent (Takara Biotechnology (Dalian) Co., Ltd.) was used to bind and transport active anti-HURP rabbit polyclonal Ab (Santa Cruz Biotechnology, Inc.) directly into the breast cancer cells. The cells (1.2×10^5^) were transfected with concentration gradients of the anti-HURP Ab (4 and 5 μg, respectively)using the Xfect Protein Transfection Reagent, according to the manufacturer’s instructions. Following incubation at 37°C for 60 minutes, the cells were harvested for the next cell viability assays. In addition, β-galactosidase was used as the control. The cells were stained with X-gal to determine the efficiency of β-galactosidase transfection using the β-Galactosidase Staining kit (Takara Bio, Inc.).

### Cell proliferation assays

Cell proliferation assays were performed using Cell Counting Kit-8 (Dojindo Laboratories, Kumamoto, Japan). The cells were plated in 96-well plates, at 1×10^5^ cells per well, and cultured in the growth medium. At the indicated time-points, the cell numbers in triplicate wells were measured at the absorbance (450 nm) of reduced WST-8 [2-(2-methoxy-4-nitrophenyl)-3-(4-nitrophenyl)-5-(2,4-disulfophenyl)-2H-tetrazolium, monosodium salt].

### Statistical analysis

The statistical analysis was conducted using SPSS software (version 19.0; SPSS, Inc., Chicago, IL, USA). The HURP normalized expression values were expressed as the mean ± standard deviation (SD) and compared using Student’s t-test. Differences between the groups were determined using the Mann-Whitney-Wilcoxon U test and Kruskal-Wallis test. P<0.05 was considered to indicate a statistically significant difference.

## Results

### HURP overexpression in human breast cancer

**Figure 1:**
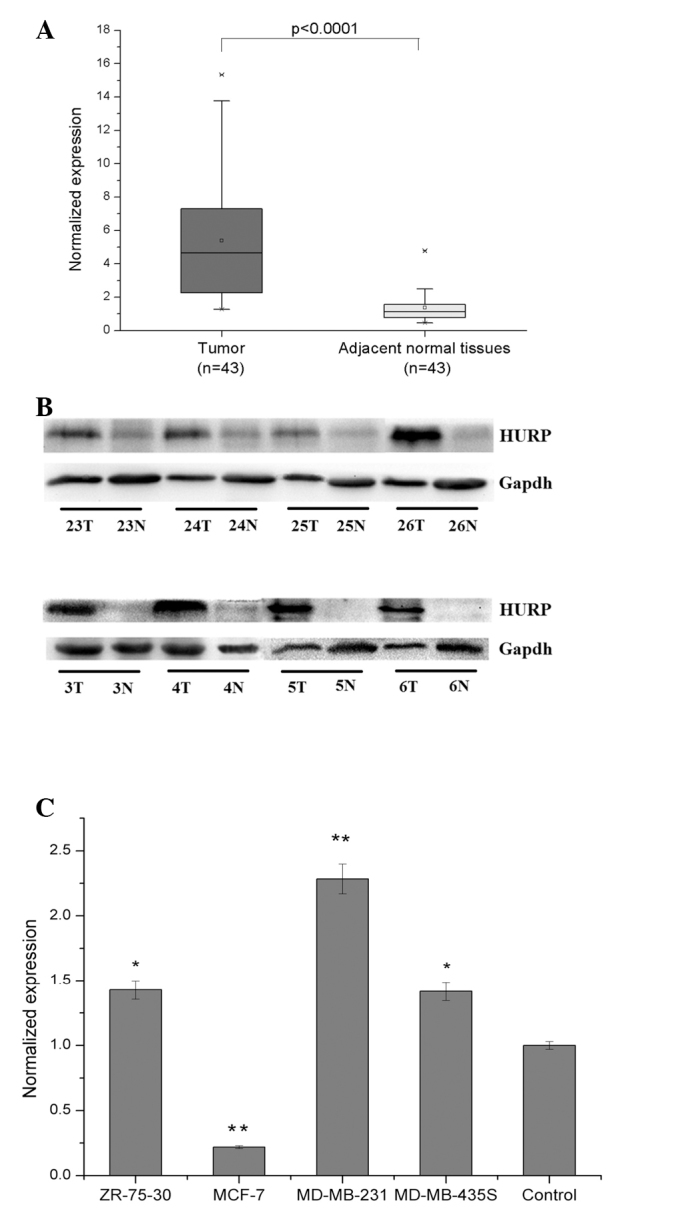
Hepatoma upregulated protein (HURP) mRNA expression in breast tumors and adjacent normal tissues. (A) Mean HURP expression in primary breast tumors (5.38±3.71; mean ± standard deviation) compared with adjacent normal tissues (1.37±0.87; P<0.0001, Student’s t-test). (B) Representative images of the HURP protein level from breast tumors (T) and adjacent normal tissues (N) were calculated by western blot analysis. The protein level of HURP was normalized to the level of glyceraldehyde-3 phosphate dehydrogenase (Gapdh) protein. (C) HURP mRNA expression in breast cancer cell lines. The values are expressed as the mean ± standard error of the mean for three independent sets of data. ^*^P<0.05 and ^**^P<0.01 vs. control group

qPCR analysis of HURP mRNA expression in 43 pairs of primary breast tumors and adjacent histologically normal tissues revealed a significantly (P<0.0001) higher expression level of HURP in the tumor tissue (n=43; 90%) compared with the normal tissue. The mean expression level of the HURP mRNA in the tumor tissues was 5.38±3.71 (mean ± SD), which was significantly higher than the mean of 1.37±0.87 in the corresponding paired adjacent normal tissues (Fig. 1A). Western blot analysis with anti-HURP Ab verified that HURP protein levels were unregulated in the human breast tumor tissues compared with the normal tissues. Representative images of western blots are shown in Fig. 1B. Significantly high levels of HURP mRNA expression were also detected in three, MDA-MB-231, MDA-MB-435S and ZR-75-30, of the four, MDA-MB-231, MDA-MD-435S, ZR-75-30 and MCF-7, human breast cancer cell lines examined (Fig. 1C). Together, these results indicate that HURP is overexpressed in human breast cancer cells that have a high proliferative and invasive ability.

### Clinicopathological significance of HURP expression in human breast cancer

To further investigate the association between HURP and breast cancer, 43 malignant tumors were analyzed. The clinicopathological factors analyzed in relation to HURP normalized expression are shown in Table I. The expression of HURP was positively correlated with the TNM staging (P=0.003). Conversely, no significant differences were observed between the age, size, estrogen receptor response, progesterone receptor response, human epidermal receptor presence, extent of lymph node metastasis or differentiation and HURP expression.

### Suppression of HURP expression in breast cancer cells inhibits proliferation

To investigate the clinical findings of HURP in breast cancer and to understand its role in carcinogenesis, *in vitro* functional studies of HURP were performed. The primary focus was whether HURP overexpression is associated with the proliferative potency of breast cancer cells, as HURP has been reported to be associated with proliferation activity in other cancers (18). The highest mRNA expression level of HURP among the MDA-MB-231, MDA-MD-435S, ZR-75-30 and MCF-7 cell lines was exhibited by MDA-MB-231 (Fig. 1C). MDA-MB-231 was also the most sensitive to HURP-specific DsiRNA transfection and had consistent stability with DsiRNA transfection. Therefore, MDA-MB-231 was selected as the representative cell line for study.

**Figure 2:**
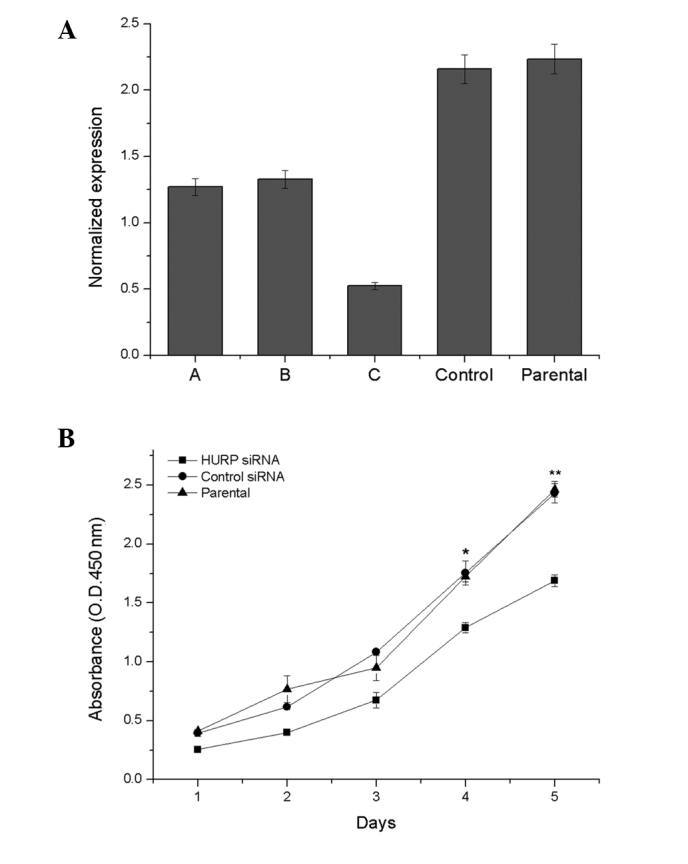
Suppression of hepatoma upregulated protein (HURP) inhibits breast cancer cell proliferation *in vitro*. (A) The HURP mRNA normalized expression levels of MDA-MB-231 cells transfected with: A, gene-specific 27mer small interfering (si)RNA duplex 1 (DsiRNA1); B, DsiRNA2; C, DsiRNA3; negative control siRNA duplex and non-transfected cells. The expression levels of the HURP mRNA were markedly suppressed in HURP DsiRNA3-transfected cells when compared with the others. (B) MDA-MB-231 cell viability was measured using a Cell Counting Kit-8 assay for 5 days. The viability of the HURP DsiRNA3-transfected cells was lower compared with the parent cells and cells transfected with the negative control siRNA duplex (^*^P<0.05 and ^**^P<0.01 vs. control group).

qPCR analysis confirmed that the HURP mRNA expression level was lower in the HURP DsiRNA3-transfected MDA-MB-231 cells compared with the MDA-MB-231 cells transfected with DsiRNA1 or DsiRNA2, the negative control siRNA duplex and the non-transfected cells (Fig. 2A). Therefore, HURP DsiRNA3 was chosen as the inhibitor. The cell proliferation analysis demonstrated that suppression of HURP by HURP DsiRNA3 significantly inhibited cell growth. HURP DsiRNA3 cells grew slower than the parent or control cells in the CCK-8 assay (Fig. 2B).

### Ab-mediated disruption of HURP function in breast cancer cells inhibits proliferation

**Figure 3:**
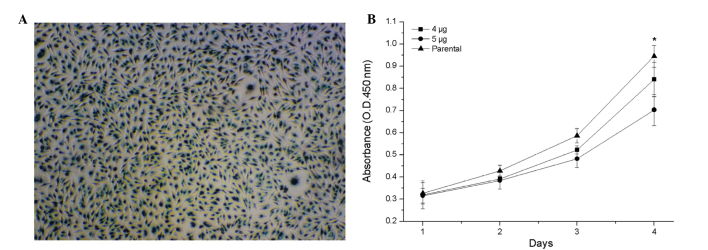
Interference in hepatoma upregulated protein (HURP) expression inhibits breast cancer cell proliferation *in vitro*. (A) MDA-MB-231 cells transfected with 2μg β-galactosidase (β-gal) using Xfect Protein Transfection Reagent. At 1 h post-transfection, the cells were assayed for β-gal activity using a β-Galactosidase Staining kit. The image was captured using an inverted microscope with ×100 magnification. The Xfect Protein Transfection Reagent displayed a markedly higher signal for β-gal. (B) MDA-MB-231 cell viability was measured using the Cell Counting Kit-8 assay for four days. The viability of the anti-HURP Ab-transfected cells was lower than that of the parent cells. The cells transfected with 5μg anti-HURP Ab grew slower than the other transfected cells (^*^P<0.05 and ^**^P<0.01).

To investigate the transient effects of HURP and the possibility of McAb or polypeptide drug treatment, active anti-HURP Ab was transfected directly into the MDA-MB-231 cells. As the control transfection, the MDA-MB-231 cells were transfected with 2μg β-galactosidase (β-gal), which revealed a high efficiency and a high amount of β-gal protein per MDA-MB-231 cell (Fig. 3A). Cell proliferation analysis revealed that anti-HURP Ab-mediated disruption of HURP activity significantly inhibited cell growth. The higher the amount of anti-HURP Ab transfected into the MDA-MB-231 cells, the slower the cells grew (Fig. 3B).

## Discussion

In the present study, the involvement of HURP in human breast cancer carcinogenesis was investigated. The results demonstrated that HURP mRNA and protein expression were significantly higher in the breast cancer tumors than in the paired normal tissues. The overexpression of HURP was also more prevalent in the breast cancer cells that exhibited increased proliferation and invasion (MDA-MB-231, MDA-MD-435S and ZR-75-30). The statistical analysis indicated that the HURP expression level was higher in the tumors with advanced-grade metastasis and was strongly associated with the tumor stage. This suggests that HURP is overexpressed in human breast cancer and that such overexpression is correlated with tumors in advanced-grade metastasis, which may be prognostic of a poor survival rate. This is the first study demonstrating the role of HURP in breast cancer progression and its association with the clinicopathological factors of the disease. In addition, these results are consistent with the previous findings that HURP is overexpressed in hepatocellular carcinoma (5), adrenocortical tumors (19) and urothelial carcinoma (20). All these studies suggest that HURP plays an important role in human cancer, particularly in tumor progression.

Through cell proliferation assays, the potential role of HURP in tumor formation and progression was determined. The present results revealed that the suppression of HURP expression by siRNA or anti-HURP Abs in breast cancer cells inhibited cell proliferation *in vitro*. These *in vitro* findings are compatible with the high expression levels of HURP observed in breast cancer tissues from patients with aggressive disease. Previous studies demonstrated that the overexpression of HURP in non-tumorigenic HEK293 cells increases their proliferative ability and transformation activity (21), in addition to enhancing the invasiveness of hepatocellular carcinoma cells (22). More recently, HURP has been demonstrated to be the direct target gene of NOTCH3, as growth inhibition in ovarian cancer cells induced by pharmacological or RNA interference-mediated NOTCH inhibition is notably prevented by the enforced expression of HURP (23). The present results are consistent with these findings, indicating that the deregulation of HURP expression, such as overexpression, in tumor cells, inhibits cell growth.

HURP is an essential component of the mitotic apparatus, which can form a complex with RanGTP and localize predominantly to the K-MTs *in vivo*. By stabilizing the MTs, HURP promotes MT polymerization and bipolar spindle formation when cells enter mitosis (7). Recent studies have demonstrated that the modulation of kinesin Kif18A function by HURP results in the regulation of chromosome congression. A higher level of HURP expression leads to increased Kif18A sequestration at the K-MTs and a chromosome congression defect is more likely to occur (24). In other studies, HURP reduced the levels of p53 in normal and cancerous cells, and is therefore indicated to act as an oncogene. Thus, suppression of HURP may interfere with the interphase dynamics of MTs, affect the growth or stability of spindle MTs and inhibit tumor growth. MT-targeting agents have made a noteworthy contribution to cancer therapy over the past 50 years and include the vinca alkaloids and taxanes, which have been used to treat a broad range of malignancies (25,26). Therefore, HURP-targeted therapy may be of potential benefit in treating breast cancer in the future. The present study attempted, for the first time, to transfect anti-HURP Abs in order for them to directly act on HURP in cancer cells. The results of the anti-HURP Ab transfection demonstrate that HURP-targeted therapy may be effective in blocking the progression of breast cancer. McAbs or polypeptide drugs, which have a more effective target area and less toxicity, will be the focus of future studies.

In conclusion, the present study found that HURP expression was significantly elevated in breast cancer tumors and that elevated HURP expression was associated with the proliferation of breast cancer and the degree of malignancy. In addition to being a tumor biomarker for prognosis, HURP may serve as a potential therapeutic drug target for human breast cancer.

## Figures and Tables

**Table I:** HURP mRNA expression and clinocopathological factors

Prognostic factors	No. of patients (n=43)	Expression of HURP, mean ± SD	P-value
Age, years			
>50	13	4.91±2.70	0.881
≤50	30	5.31±3.86	
Size, mm			
<20	19	5.67±3.77	0.490
20–50	20	4.97±3.46	
>50	4	3.56+2.07	
Lymph node metastasis			
Absent	27	4.72±3.39	0.125
Present	16	6.51±4.06	
ER			
Positive	34	5.22±3.72	0.798
Negative	9	5.03±2.62	
PR			
Positive	33	5.32±3.74	0.890
Negative	10	4.69±2.67	
HER			
Positive	39	5.18±3.62	0.643
Negative	4	5.17±1.96	
Differentiation			
G1	2	2.28±0.82	0.253
G2	17	5.15±4.36	
G3	19	5.75±3.65	
pTNM			
I	5	2.32±0.58	0.017^a^
II	29	5.09±3.36	
III	9	8.05±4.29	

Mann-Whitney-Wilcoxon U test and Kruskal-Wallis test,

a P≤0.05.

HURP, hepatoma upregulated protein; pTNM, pathological tumor-node-metastasis; ER, estrogen receptor; PR, progesterone receptor; HER, human epidermal receptor; SD, standard deviation.
